# Mechanisms of Adipose Tissue Metabolism in Naturally Grazing Sheep at Different Growth Stages: Insights from mRNA and miRNA Profiles

**DOI:** 10.3390/ijms26073324

**Published:** 2025-04-02

**Authors:** Xige He, Yunfei Han, Lu Chen, Yueying Yun, Yajuan Huang, Gerelt Borjigin, Buhe Nashun

**Affiliations:** 1 Inner Mongolia Key Laboratory for Molecular Regulation of the Cell, Inner Mongolia University, Hohhot 010070, China; 2 State Key Laboratory of Reproductive Regulation and Breeding of Grassland Livestock, School of Life Sciences, Inner Mongolia University, Hohhot 010040, China; 3 College of Food Science and Engineering, Inner Mongolia Agricultural University, Hohhot 010018, China; hanyunfei122@163.com (Y.H.); chenluuu0618@163.com (L.C.); huangyajuan2008@163.com (Y.H.); bor_gerelt07@imau.edu.cn (G.B.); 4 Inner Mongolia Academy of Agricultural and Animal Husbandry Sciences, Hohhot 010031, China; 5 School of Life Science and Technology, Inner Mongolia University of Science and Technology, Baotou 014010, China; yunyueying1999@163.com

**Keywords:** fat deposition, miRNA, mRNA, Ovis aries, subcutaneous adipose tissue

## Abstract

Adipose tissue metabolism plays a crucial role in sheep meat quality and the optimization of adipose tissue utilization. To reveal the molecular mechanisms of adipose tissue metabolism during growth in naturally grazing sheep, we investigated the mRNA and miRNA profiles in subcutaneous adipose tissue (SAT) from naturally grazing Sunit sheep at 6, 18, and 30 months of age (Mth-6, Mth-18, and Mth-30). We identified 927 differentially expressed (DE) genes and 134 DE miRNAs in the SAT of sheep at different growth stages. Specifically, the expressions of *ACACA*, *FASN*, *DGAT2*, *GPAM*, *SCD*, *ELOVL6*, *HSD17B12*, *TECR*, *PKM*, *TKT*, *PCK1*, *CD44*, and *THBS2S* genes were significantly upregulated in Mth-18 and Mth-30 compared to that in Mth-6. These genes promoted fatty acid synthesis, triglyceride synthesis, gluconeogenesis, and extracellular matrix–receptor interaction and decreased glycolysis, leading to increased adipocyte proliferation and fat deposition. Notably, our findings suggested that the reduced activity of the AMPK signaling pathway may be regulated by *CAMKK2* and *PP2A* during sheep growth. Furthermore, our results revealed several DE miRNAs, *mml-miR-320b*, *chi-miR-1388-3p*, *bta-miR-6715*, *oar-miR-143*, and *miR-424*, that potentially influence fat metabolism. Overall, this study provides a theoretical basis and new insights into the molecular mechanisms of adipose tissue metabolism during growth in naturally grazing sheep.

## 1. Introduction

The *Mongolian sheep*, a superior local breed, with the highest production in China exhibits remarkable adaptability to the extreme environmental conditions of the Mongolian Plateau, including cold climate, food scarcity, and high altitudes. This adaptation is associated with its abundant adipose tissue [1]. Adipose tissue, an essential and dynamic endocrine organ [2], which is distributed in various parts of the sheep body, including the subcutaneous, tail, visceral, intramuscular, and intermuscular regions. Among these, subcutaneous adipose tissue (SAT) exhibits the widest distribution among these tissues and is closely associated with carcass characteristics, such as intramuscular fat content, tenderness, flavor, and protection against cold shortening and drip loss during cooling of the carcass [3,4]. Similar to the functions of intermuscular and intramuscular fat in meat quality attributes, SAT affects the taste, flavor, and nutritional value of edible meat [5]. Furthermore, adipose tissue serves as a valuable resource for industries producing soaps, lipsticks, and shortenings. Therefore, investigating adipose tissue traits is imperative to enhance the meat quality and optimize its utilization.

SAT metabolism in *Mongolian sheep* is a multifaceted trait shaped by a confluence of factors, including genetics, climate, grazing periods, and forage composition. Zhang et al. [2] showed that grazing *Mongolian sheep* depend on the expansion of both interscapular and subcutaneous white adipose tissue for acclimatization to the cold environment of the Mongolian Plateau. Another study revealed that cold exposure triggers UCP1-dependent thermogenesis and activates calcium and cAMP signaling pathways within adipose tissues in lambs [6]. Particularly, the seasonal shift between dry and green grass periods induces significant variations in the amino acid and fatty acid composition of adipose tissue [7]. Thus, climate exerts a dominant influence on the growth of Mongolian sheep, encompassing its impact on both the environmental conditions and pasture quality.

The adipose tissues of sheep exhibit remarkable plasticity, enabling efficient energy storage and mobilization through intricate cellular and molecular mechanisms. Fat metabolism primarily depends on lipid uptake, lipogenesis, and fatty acid oxidation [8]; glycolysis/gluconeogenesis, protein metabolism, and the citrate cycle also provide substrates and energy for fat metabolism [9]. Key signaling pathways, such as the PPAR, MAPK, and AMPK, can also activate or inhibit the expression of related genes and play a regulatory role in fat metabolism [10,11]. Furthermore, several miRNAs, including *miR-152*, *miR-320b*, *miR-6715*, *miR-143*, and *miR-196b*, act by inhibiting target gene translation or promoting mRNA degradation, to regulate adipocyte proliferation and deposition [12,13,14,15]. Adipose tissue metabolism varies due to breed, anatomical site, environmental temperature, exercise, grazing periods, and age, with varied metabolic mechanisms [2,7,16,17].

Typically, companies prefer slaughtering sheep under the age of one year (especially approximately six-month-old lambs) for optimal commercial value. However, local inhabitants tend to slaughter sheep older than one year or even three years for consumption purposes. Our previous studies have revealed that during the growth of sheep under natural grazing conditions, SAT deposition was increased via adipocyte proliferation rather than hypertrophy; free fatty acids and fatty acids in lipids were elongated; and acylcarnitines, fatty acid amides, aspartic acid, acetic acid, and phosphocholine were altered substantially [18]. However, the molecular mechanisms of these processes remain unclear. In this study, SAT samples were collected from 6-month-old, 18-month-old, and 30-month-old naturally grazing Mongolian sheep in October of the same year. Then, we characterized the mRNA and miRNA profiles across different groups and conducted miRNA–mRNA-targeted regulatory networks. The results were integrated with the analysis of fat thickness, adipocyte morphology, fatty acids, and metabolomics data, to identify key differentially expressed (DE) mRNAs and miRNAs, thereby elucidating the molecular mechanisms underlying adipose tissue development. This study offers valuable insights for optimizing animal breeding strategies and enhancing sheep meat quality.

## 2. Results

### 2.1. mRNA Sequencing Analysis

#### 2.1.1. Overview of mRNA Sequencing Data

To investigate the potential function of RNAs in sheep SAT across three different growth stages (6, 18, and 30 months), we constructed nine libraries (three biological replicates each for Mth-6, Mth-18, and Mth-30). In total, 812,208,754 raw reads were generated, and more than 94.4% of them were observed to be valid reads after the low-quality bases were filtered out. On average, approximately 91.9% of the valid reads from each library were mapped to the sheep reference genome (Table S3). Overall, these high mapping rates and quality metrics confirmed the robustness of our RNA sequencing data, enabling reliable downstream analyses.

#### 2.1.2. Differential Expression Analysis of Genes

**Figure 1:**
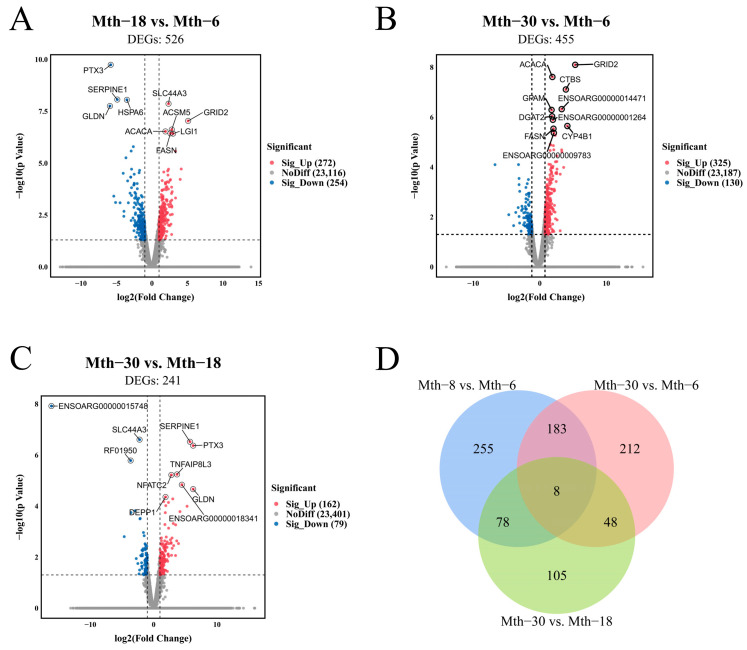
Differential expression analysis of genes. (**A**): Mth-18 vs. Mth-6; (**B**): Mth-30 vs. Mth-6; (**C**): Mth-30 vs. Mth-6. (**D**): Venn diagram showing the overlap of DEGs among Mth-18 vs. Mth-6, Mth-30 vs. Mth-6, and Mth-30 vs. Mth-6.

We compared the miRNA and mRNA expression levels by pairwise comparisons (Mth-18 vs. Mth-6, Mth-30 vs. Mth-6, and Mth-30 vs. Mth-18). In total, 927 DEGs were identified at different growth stages, including *ACACA*, *FASN*, *DGAT2*, *GPAM*, and *SCD*. DEGs were observed in Mth-18 vs. Mth-6, including 272 and 254 DEGs with upregulated and downregulated expression, respectively (Figure 1A). A total of 455 DEGs were observed in Mth-30 vs. Mth-6, including 325 and 130 DEGs with upregulated and downregulated expression, respectively (Figure 1B). In Mth-30 vs. Mth-18, 241 DEGs were observed including 162 and 79 DEGs with upregulated and downregulated expression, respectively (Figure 1C). The number of DEGs was the lowest in the Mth-30 vs. Mth-18 comparison. Eight overlapping DEGs were identified between the three comparison groups and 183 overlapping DEGs were identified between Mth-18 vs. Mth-6 and Mth-30 vs. Mth-6 (Figure 1D). These results indicate that the mRNA profiles of SAT in sheep differed at different growth stages, with significant variations observed between Mth-6 and Mth-18, as well as Mth-30. Additionally, numerous DEGs were associated with fat metabolism.

#### 2.1.3. Functional Annotation and Enrichment Analysis of DEGs

**Figure 2:**
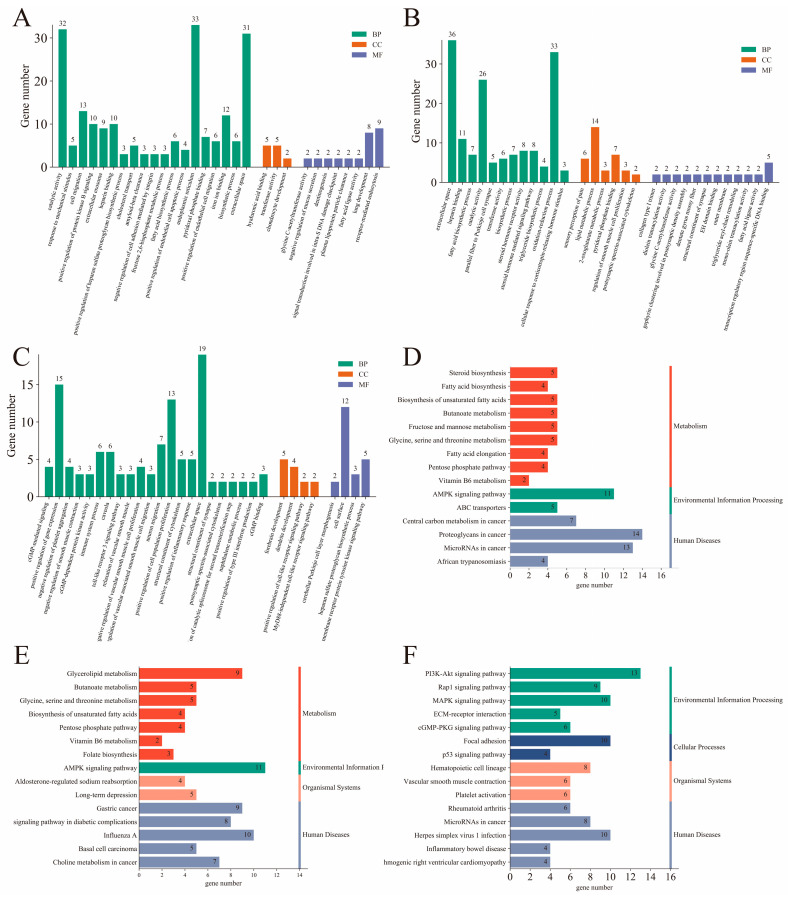
The enrichment analysis of DEGs. Top 30 GO function enrichment analysis of DEGs ((**A**): Mth-18 vs. Mth-6; (**B**): Mth-30 vs. Mth-6; (**C**): Mth-30 vs. Mth-6)); top 15 KEGG pathways enrichment analysis of DEGs ((**D**): Mth-18 vs. Mth-6; (**E**): Mth-30 vs. Mth-6; (**F**): Mth-30 vs. Mth-6). MF: molecular function; BP: biological process; CC: cellular component.

**Figure 3:**
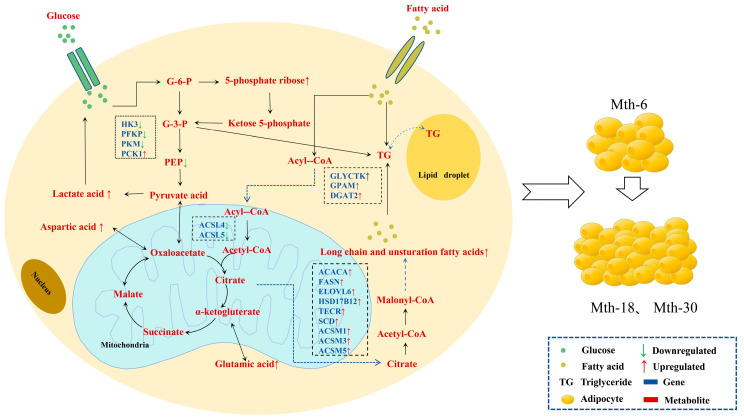
Mechanisms of subcutaneous adipose tissue metabolism.

**Figure 4:**
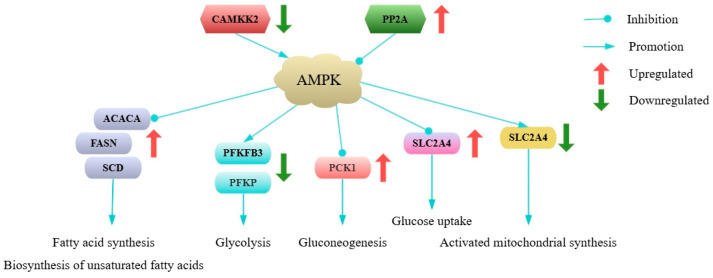
Changes in the expression of genes related to the AMPK signaling pathway.

To understand the functions of DEGs, functional annotation and enrichment analyses were performed using the GO and KEGG databases. In total, 26,247 genes were annotated. These genes had 18,361 and 6412 annotations in the GO and KEGG databases, respectively. We further analyzed the DEGs for enrichment in GO and KEGG. GO terms were determined using three functions: biological processes (BP), cellular components (CC), and molecular functions (MF). In the 3 different stages, the DEGs were significantly enriched for 1123 GO terms, and the 30 most significantly enriched GO terms are presented in a histogram (Figure 2A–C). Among the 3 comparison groups, 11 GO terms were enriched between 2 or more comparison groups. These included heparin binding, catalytic activity, transferase activity, pyridoxal phosphate binding, glycine C-acetyltransferase activity, structural constituent of synapse, and fatty acid ligase activity in the MF category; fatty acid biosynthetic process and biosynthetic process in the BP category; and extracellular space and postsynaptic spectrin-associated cytoskeleton in the CC category. KEGG pathway analysis of the DEGs was also performed; the top 15 KEGG pathways are shown in a histogram (Figure 2D–F). Among these, biosynthesis of unsaturated fatty acids; butanoate metabolism; AMPK signaling pathway; glycine, serine, and threonine metabolism; pentose phosphate pathway; and vitamin B6 metabolism were enriched in Mth-18 vs. Mth-6 and Mth-30 vs. Mth-6. Table 1, Table 2 and Table 3 present the expression trends of genes and pathways related to fat metabolism in different comparison groups. We found that the expressions of most of the genes related to fatty acid synthesis, lipid synthesis, the pentose phosphate pathway, and amino acid metabolism were upregulated. The expressions of all DEGs related to extracellular matrix (ECM)–receptor interaction were upregulated, and there were many DEGs enriched in the AMPK signaling pathway. Based on these genes and their enrichment pathways, we constructed maps of the metabolic mechanisms underlying the growth of Mongolian sheep (Figure 3 and Figure 4). These findings suggest that alterations in lipid and glucose metabolism-related genes in Mth-18 and Mth-30 facilitated SAT deposition compared to that in Mth-6; the attenuated activity of the AMPK signaling pathway contributed to SAT accumulation.

### 2.2. miRNA Sequencing Analysis

#### 2.2.1. Overview of miRNA Sequencing Data

A total of 9 small RNA libraries were constructed and sequenced, producing 51,204,498 valid reads. The average gene–mapping ratio for each sample was 41.94% (Table S4). The length distribution showed that most of these reads were in the range of miRNAs, from 21 to 23 nt (Figure S1). In total, 1573 miRNAs were obtained from the SAT of sheep at 3 different growth stages, of which 1099 were known miRNAs and 474 were novel miRNAs.

#### 2.2.2. Differential Expression Analysis of miRNAs

**Figure 5:**
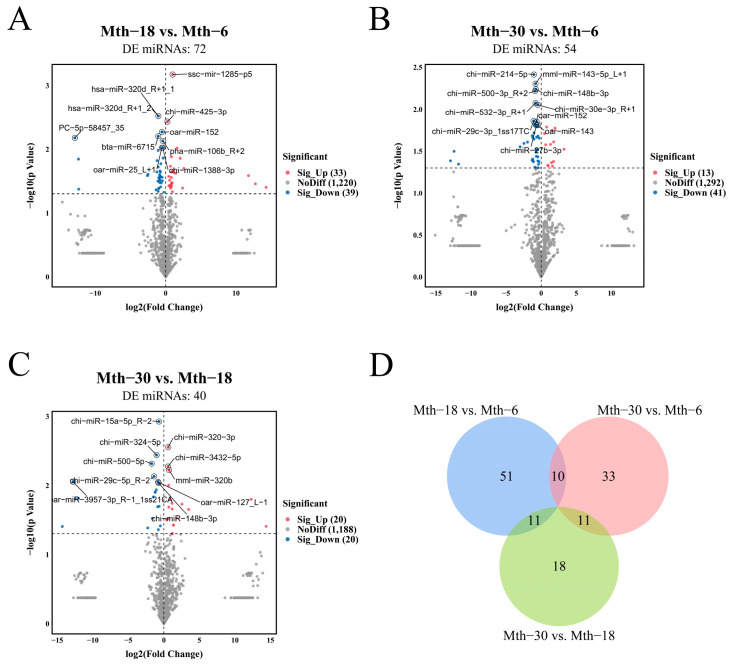
Differential expression analysis of miRNAs. (**A**): Mth-18 vs. Mth-6; (**B**): Mth-30 vs. Mth-6; (**C**): Mth-30 vs. Mth-6. (**D**): Venn diagram showing the overlap of DE miRNAs among Mth-18 vs. Mth-6, Mth-30 vs. Mth-6, and Mth-30 vs. Mth-6.

In the 3 comparison groups, 134 DE miRNAs (including 13 novel miRNAs) were identified (Figure 5). Further, 72 DE miRNAs (33 and 39 with upregulated and downregulated expressions, respectively), including 9 novel miRNAs, were obtained in Mth-18 vs. Mth-6. In Mth-30 vs. Mth-6, 54 DE miRNAs (13 and 41 with upregulated and downregulated expressions, respectively), including 4 novel miRNAs, were identified. In Mth-30 vs. Mth-18, 40 miRNAs (20 and 20 with upregulated and downregulated expressions, respectively) were DE, and 1 novel miRNA was detected. The number of DE miRNAs was the least in Mth-30 vs. Mth-18. These results also indicated that the mRNA profiles of SAT in sheep differed at different growth stages, with significant variations observed between Mth-6, Mth-18, and Mth-30, which was consistent with the DEG analysis results.

#### 2.2.3. Enrichment Analysis of Target Genes for DE miRNAs

**Figure 6:**
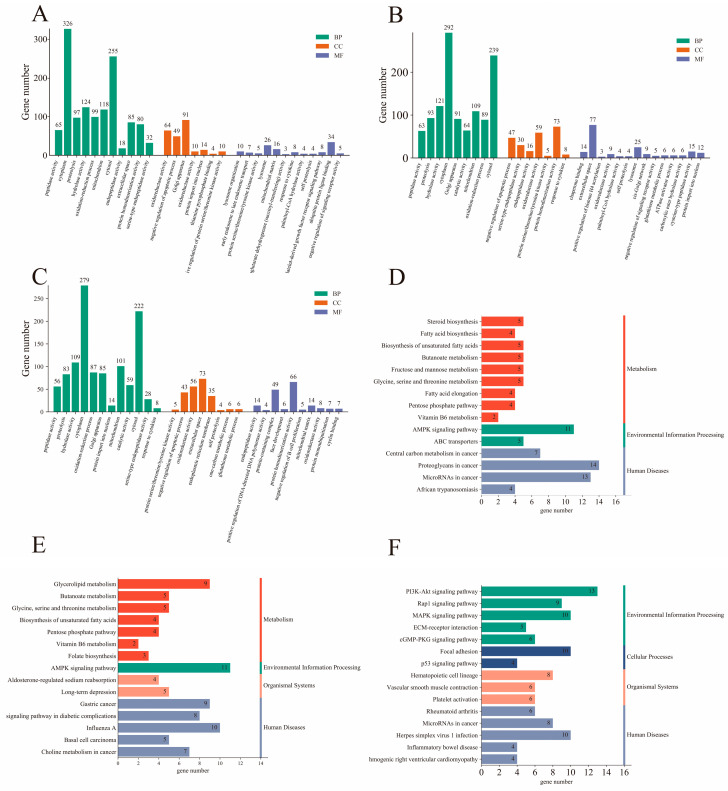
The enrichment analysis of DE miRNAs. Top 30 GO function enrichment analysis of DEGs ((**A**): Mth-18 vs. Mth-6; (**B**): Mth-30 vs. Mth-6; (**C**): Mth-30 vs. Mth-6)); top 15 KEGG pathways enrichment analysis of DEGs ((**D**): Mth-18 vs. Mth-6; (**E**): Mth-30 vs. Mth-6; (**F**): Mth-30 vs. Mth-6). MF: molecular function; BP: biological process; CC: cellular component.

To explore the function of DE miRNAs, GO function and KEGG pathway enrichment analyses of the target genes were performed. The 30 most significantly enriched GO terms are presented in a histogram (Figure 6A–C). Nineteen GO terms were co-enriched in the three comparison groups, including negative regulation of apoptotic process, extracellular space, oxidoreductase activity, and glutathione metabolic process. Palmitoyl-CoA hydrolase activity, lysosomes, and negative regulation of signaling receptor activity were enriched in Mth-18 vs. Mth-6 and Mth-30 vs. Mth-6. The mitochondrial matrix was enriched in Mth-18 vs. Mth-6 and Mth-30 vs. Mth-18. Catalytic activity and glutathione metabolic process were enriched in Mth-30 vs. Mth-6 and Mth-30 vs. Mth-18. The top 15 KEGG pathways are shown in the histograms (Figure 6D–F). Twelve KEGG pathways were enriched in the three comparison groups; of these, fatty acid elongation, the IL-17 signaling pathway, and the AMPK signaling pathway were more relevant to lipid metabolism.

### 2.3. Integrated miRNA–mRNA Interaction Analysis

**Figure 7:**
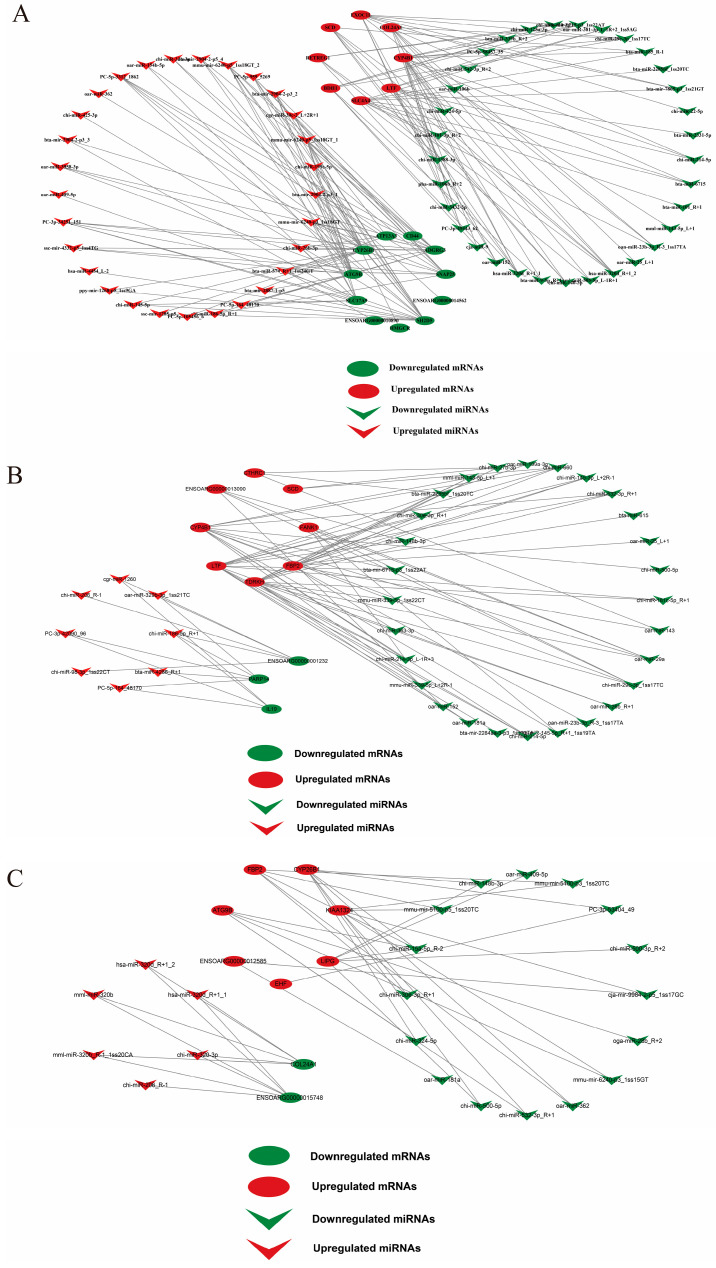
The regulatory network of miRNA–mRNAs. (**A**): Mth-18 vs. Mth-6; (**B**): Mth-30 vs. Mth-6; (**C**): Mth-30 vs. Mth-6. Green ellipses represent downregulated mRNAs; red ellipses represent upregulated mRNAs; green V-shape represents downregulated miRNAs; red V-shape represents upregulated miRNAs.

To elucidate the regulatory relationship between DE miRNAs and their target genes, we constructed a network of DE miRNAs that were negatively correlated with the DEGs (Figure 7). We observed that several DE miRNAs targeted DEGs associated with lipid metabolism, such as *SCD*, *BDH1*, *CYP4B1*, *IL19*, *COL24A1*, *FBP2*, and *SLC4A4*. Six DE miRNAs targeted *SCD*, seven targeted *BDH1*, and thirty-one targeted *CYP4B1*, including four DE miRNAs (*bta-mir-6715-p3_1ss22AT*, *chi-miR-214-5p*, *chi-miR-29c-3p_1ss17TC*, and *mml-miR-143-5p_L+1*), the expressions of which were downregulated in Mth-18 vs. Mth-6 and Mth-30 vs. Mth-6. *FBP2* expression was significantly upregulated in Mth-30 vs. Mth-6 and in Mth-30 vs. Mth-18, and 14 DE miRNAs targeted *FBP2*, of which the expression of *chi-miR-30e-3p_R+1* was downregulated in Mth-30 vs. Mth-6 and Mth-30 vs. Mth-18. Four DE miRNAs targeted IL19. Moreover, we found that the expression of the target gene *COL24A1* was upregulated in Mth-18 vs. Mth-6, with eight DE miRNAs targeting *COL24A1*. However, the expression was downregulated in Mth-30 vs. Mth-18, with five DE miRNAs targeting *COL24A1*. Among these, the expressions of *hsa-miR-320d_R+1_1* and *hsa-miR-320d_R+1_2* were downregulated in Mth-18 vs. Mth-6 and upregulated in Mth-30 vs. Mth-18; we thus hypothesized that *miR-320d* plays an important role in regulating *COL24A1*. The expressions of 11 DE miRNAs were upregulated in Mth-18 vs. Mth-6 and targeted *SLC4A4*, including *bta-miR-6715* and *chi-miR-1388-3p*.

### 2.4. Validation Using RT-qPCR

**Figure 8:**
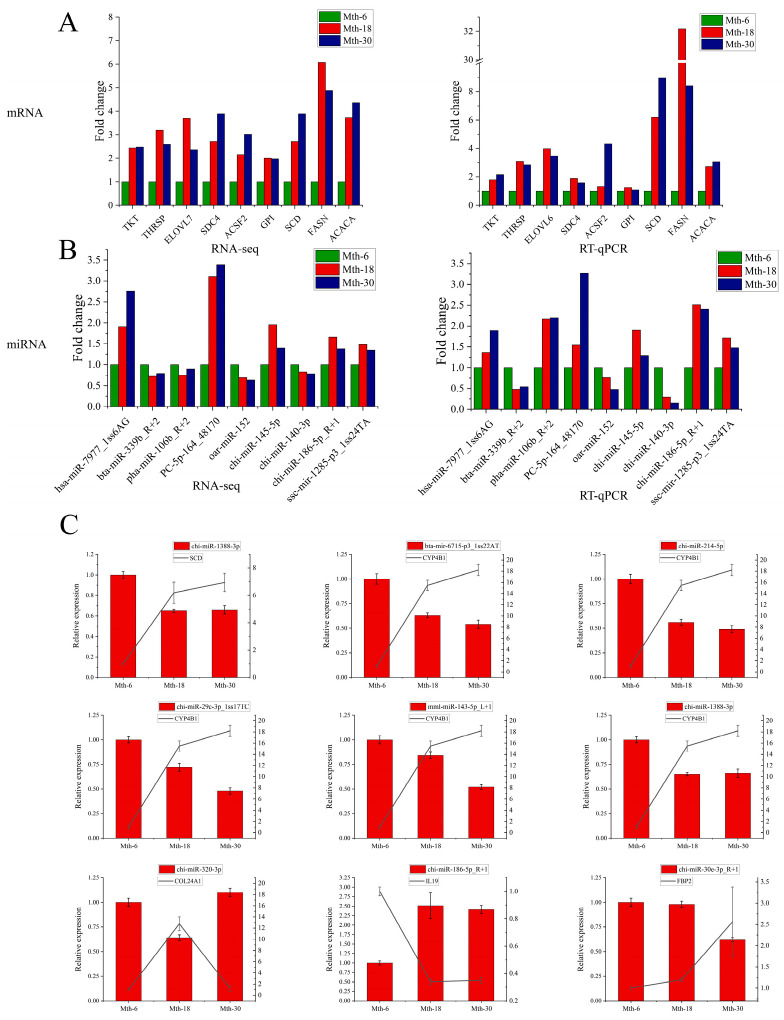
Validation using RT-qPCR. (**A**): Validation of mRNA-seq data by RT-qPCR; (**B**): validation of miRNA-seq data by RT-qPCR; (**C**): RT-qPCR validation of miRNAs and target genes.

To validate the sequencing data, nine targets for each of these groups were randomly selected for determining their expression levels using RT-qPCR (Figure 8A,B). The expression trends observed using RT-qPCR were consistent with those based on the sequencing data, indicating that the sequencing results were reliable. Using integrated miRNA–mRNA interaction analysis, we found that several DE miRNAs affect SAT metabolism by binding to target genes. Multiple target pairs were screened and validated using qRT-PCR to determine the relationship between DE miRNAs and their target genes. The results revealed a negative correlation between miRNAs and their targets (Figure 8C).

## 3. Discussion

Adipose tissue metabolism is important for sheep meat quality and for optimizing its utilization. Previous studies have revealed variations in fat thickness, adipocyte morphology, and SAT metabolites in Sunit sheep at different growth stages [18]. With the growth of sheep, an increase in SAT deposition was observed owing to adipocyte proliferation. Moreover, the carbon chains of fatty acids and lipids were elongated from Mth-6 to Mth-18 and Mth-30. Lipid compounds co-regulate SAT metabolism with non-lipid compounds. Both mRNAs and miRNAs are important regulators of adipose tissue development. Therefore, to explore the molecular mechanism of adipose tissue metabolism, high-throughput sequencing technology was used to characterize the mRNA and miRNA expression profiles of the SAT from Mongolian sheep at Mth-6, Mth-18, and Mth-30 in this study.

### 3.1. Effects of Gene Expression Changes on Adipose Tissue Metabolism

In the present study, 526, 455, and 241 DEGs, respectively, were identified in the Mth-18 vs. Mth-6, Mth-30 vs. Mth-6, and Mth-30 vs. Mth-18 comparisons. The GO and KEGG enrichment analyses revealed that these DEGs primarily participated in key metabolic pathways, including glycerolipid metabolism, fatty acid metabolism, biosynthesis of unsaturated fatty acids, fatty acid elongation, glycolysis/gluconeogenesis, and the AMPK signaling pathway. Notably, previous studies have indicated that *FASN*, *ACACA*, *DGAT2*, *DGKA*, and *GPAM* catalyze crucial reactions in animal lipid metabolism. Specifically, acetyl-CoA carboxylase alpha (*ACACA*) and fatty acid synthase (*FASN*) are pivotal for de novo fatty acid synthesis [19]. FASN also participates in the adipogenesis-associated de novo synthesis of long-chain fatty acids facilitated by malonyl-CoA [20]. Moreover, the *ELOVL* gene family plays a crucial role in fatty acid elongation, and it is a vital regulator of cellular lipid composition [21]. Further, *DGAT2*, *DGKA*, and *GPAM* play important roles in regulating cellular triglyceride and phospholipid levels [22,23,24]. In this study, the expressions of *FASN*, *ACACA*, *DGAT2*, *DGKA*, *ELOVL6*, and *GPAM* were upregulated in Mth-18 and Mth-30 compared to those in Mth-6, suggesting enhanced fatty acid and lipid biosynthesis, ultimately leading to increased fat deposition.

Fatty acyl-CoA synthetases facilitate the synthesis of FAs into acyl-CoA [25], which acts as a substrate for both lipid synthesis and fatty acid oxidation. Fatty acyl-CoA synthetases encompass short-chain fatty acyl-CoA synthetases, medium-chain fatty acyl-CoA synthetases (*ACSMs*), and long-chain fatty acyl-CoA synthetases (*ACSLs*). Notably, the expressions of *ACSM1*, *ACSM3*, and *ACSM5* were upregulated in Mth-18 and Mth-30 compared to those in Mth-6, while *ACSL4* and *ACSL5* were downregulated. The alterations in carbon chains of fatty acids and lipids during the growth and development of sheep may primarily stem from variations in these genes. Additionally, genes involved in fatty acid elongation and desaturation, namely *TECR*, *SCD*, and *HSD17B12*, also upregulated in Mth-18 and Mth-30, suggesting their crucial roles in modulating lipid profiles [26,27,28].

Glycolysis/gluconeogenesis occurs upstream of de novo fatty acid synthesis and supplies substrates for fatty acid and triglyceride synthesis. Specifically, Mth-18 and Mth-30 showed downregulation of key glycolytic enzymes, including hexokinase, phosphofructokinase platelet-type, and pyruvate kinase [29,30,31]. Conversely, gluconeogenic enzymes, phosphoenolpyruvate carboxykinase (*PCK*), and fructose-1,-6-bisphosphatase (*FBP*) were upregulated in these groups [32]. Tang et al. [33] suggested that *PCK1* is overexpressed upon the activation of gluconeogenesis and suppression of glycolysis pathways. These findings suggest that weakened glycolysis is accompanied by enhanced gluconeogenesis with sheep growth, which facilitates fat deposition in Mth-18 and Mth-30.

The AMPK system senses cellular energy status [34,35]. *CAMKK2* activates the AMPK signaling pathway, which is inhibited by *PP2A* [36]. In this study, the expressions of *CAMKK2* and *PP2A* were significantly downregulated and upregulated, respectively, in Mth-18 and Mth-30 compared to those in Mth-6. We thus hypothesize that as sheep grow, AMPK signaling pathway activity is suppressed via the regulation of *CAMKK2* and *PP2A* expression. These results may promote synthesis pathways, such as fatty acid synthesis, unsaturated fatty acid synthesis, gluconeogenesis, and glucose uptake, while inhibiting catabolic pathways, such as glycolysis [34,35], and eventually influence the degree of SAT deposition. However, the precise roles of *CAMKK2* and *PP2A* in modulating the AMPK signaling pathway within sheep adipose tissue necessitate further experimental validation.

In addition to metabolic pathways, the expressions of DEGs (*CD44*, *THBS2S*, *CD61*, *ITGA5*, and *RELN*) involved in ECM–receptor interactions were upregulated in Mth-30 compared to those in Mth-18. The ECM microenvironment plays a crucial role in adipose tissue biology by influencing various aspects, such as adipocyte differentiation, remodeling, and functionality. Moreover, the ECM actively participates in adipose tissue remodeling by modulating cell–matrix interactions, signal transduction, and the release of bioactive molecules [37]. Our previous study revealed that the number of adipocytes increased significantly with the growth of the sheep and may also be associated with increased expression of these genes [18].

### 3.2. Effects of miRNA Expression Changes on Adipose Tissue Metabolism

The expressions of more than 30% of genes in eukaryotic organisms are potentially regulated by miRNAs, which also play an important role in regulating fat deposition [38]. In this study, we identified 1573 miRNAs, including 1099 known miRNAs and 474 novel miRNAs. Further, 72, 54, and 40 DE miRNAs were obtained in the Mth-18 vs. Mth-6, Mth-30 vs. Mth-6, and Mth-30 vs. Mth-18 comparisons, respectively, indicating less variation in the miRNA profiles of sheep after one year of age. This may be attributed to the environmental conditions experienced by the sheep during their growth period. Mth-6 was under warm environmental conditions, leading to a sustained increase in fat deposition. Whereas Mth-18 and Mth-30 experienced alternating hot and cold spells resulting in fat degradation and regeneration. The two older groups went through these environmental changes that may have influenced gene transcription.

GO and KEGG analyses indicated that the target genes of DE miRNAs participate extensively in fat deposition-related pathways, such as fatty acid elongation, the IL-17 signaling pathway, the AMPK signaling pathway, and the extracellular space. Network analysis highlighted key miRNAs, including *mml-miR-320b*, *chi-miR-1388-3p*, *bta-miR-6715*, *oar-miR-143*, and *miR-424*, previously implicated in fat deposition [13,39,40,41]. Further, hsa-*miR-320d_R+1_1* and *hsa-miR-320d_R+1_2* were associated with multiple target genes involved in fatty acid metabolism and adipogenesis, including *CYP4B1*, *COL24A1*, *RETREG1*, *SCD*, and *LTF*. Wang et al. [13] also demonstrated that *miR-320d* may directly affect tail fat deposition by regulating *SCD* gene expression. We found *chi-miR-1388-3p* was also linked to *CYP4B1*, *SLC4A4*, and *SCD* and that the role of *chi-miR-1388-3p* may be similar to that of *miR-320d*. In this study, *chi-miR-1388-3p* expression was significantly downregulated in Mth-18 vs. Mth-6 but did not change significantly in Mth-30 vs. Mth-18, suggesting that this gene may play a crucial role in promoting fat deposition from Mth-6 to Mth-18. Furthermore, *bta-mir-6715-p3_1ss22AT* was linked to *BDH1* and *CYP4B1*, and *bta-miR-6715* was linked to *SLC4A4* and *CYP4B1*, both of which play crucial roles in regulating fatty acid metabolism and adipogenesis by modulating the expression of their target genes. Previous studies have also demonstrated the potential impact of *miR-6715* on sheep tail fat accumulation because of its effects on adipocytes [13]. Additionally, Yu et al. [39] revealed the significant expression of *miR-6715* in bovine precursor adipocytes. Additionally, the high expression of *miR-143* and *miR-424* in Mth-6 suggest their potential role in promoting adipocyte proliferation, which is consistent with their reported functions in bovine and porcine adipocyte differentiation [40,41]. Therefore, we hypothesize that *miR-143* and *miR-424* may also promote adipocyte proliferation at Mth-6. In conclusion, using database matching and expression analysis, we identified several DE miRNAs that regulate adipogenesis and deposition by targeting multiple genes. Notably, a single miRNA can target multiple genes, whereas a single gene can be targeted by numerous miRNAs. Given the complexity of gene regulation, further evaluation of the precise regulatory mechanisms of these candidate genes is needed through cell experiments. However, the study’s limitations, including a relatively small number of biological replicates leading to insufficient statistical power, make it challenging to detect subtle differences. Future studies should increase the number of biological replicates, apply stricter differential expression thresholds, and validate key gene expression at the protein level to strengthen the data’s persuasiveness and reliability.

## 4. Materials and Methods

### 4.1. Sample Collection

The samples were obtained from castrated (within 30 d of birth) rams during three different growth stages: 6 (Mth-6, n = 3, average weight: 29.43 ± 0.90 kg), 18 (Mth-18, n = 3, average weight: 48.57 ± 1.32 kg), and 30 (Mth-30, n = 3, average weight: 56.97 ± 1.71 kg) months of age. All Mongolian sheep (Sunit sheep) were selected from the same herd and raised under natural grazing conditions in the Xilingol grasslands of the Sunit banner, Inner Mongolia. The primary forage species and their nutritional composition were as previously described by Bao et al. [42]. During this feeding process, all the sheep were fed only forage and were not given any supplemental concentrated feed. Three groups of sheep were born in April across different years (with a one-year interval between each group). All sheep were slaughtered in October of the same year (Year Mth-6 was born). Following slaughter at the local abattoir, the SAT (backfat at the 12–13th rib) was sampled, frozen in liquid nitrogen, and stored at −80 °C.

### 4.2. RNA Extraction

RNA extraction, library construction, and sequencing (RNA-Seq) were performed by Hangzhou Lianchuan Biotechnology Co., Hangzhou, China. The total RNA from Sunit sheep SAT at different growth stages was isolated and purified using TRIzol reagent (Invitrogen, Carlsbad, CA, USA) following the manufacturer’s instructions. The Agilent 2100 bioanalyzer and NanoDrop ND-1000 (NanoDrop, Wilmington, DE, USA) were used to analyze RNA quantity and purity. Only samples exhibiting an RNA integrity number (RIN) exceeding 7.0 were deemed suitable for subsequent library construction and sequencing.

### 4.3. mRNA Sequencing and Data Analysis

Approximately 5 µg of the total RNA was used to deplete ribosomal RNA using the Epicenter Ribo-Zero Gold Kit (Illumina, San Diego, CA, USA) as per the manufacturer’s instructions. The remaining RNA fragments were then reverse transcribed to form a final complementary DNA (cDNA) library using an RNA-Seq library preparation kit (Illumina, San Diego, CA, USA) according to the manufacturer’s protocol. Finally, paired-end sequencing was performed on an Illumina HiSeq 4000 according to the manufacturer’s protocol. FastQC 0.10.1 (http://www.bioinformatics.babraham.ac.uk/projects/fastqc/ (accessed on 10 December 2019)) software was used to verify sequence quality. Adaptor contamination, low-quality bases, and undetermined bases in the raw data were removed using Cutadapt 1.10. The clean reads were mapped onto the sheep genome (Ovis aries v96) using Bowtie2 2.3.5 and Tophat 2.1.0, and the mapped reads were assembled using StringTie 1.3.0 software. The mRNA expression levels were calculated as fragments per kilobase of the exon model per million mapped reads. Differentially expressed genes (DEGs) were selected based on |log2 (fold change)| >1 and *p* < 0.05 using the R package Ballgown. Further, DEGs were subjected to Gene Ontology (GO) and Kyoto encyclopedia of genes and genomes (KEGG) enrichment analyses. Statistical significance was set at *p* < 0.05.

### 4.4. miRNA Sequencing and Data Analysis

Approximately 1 µg of the total RNA was used for small RNA library construction with TruSeq small RNA sample prep kits (Illumina, San Diego, CA, USA), and single-end sequencing (36 or 50 bp) was performed on an Illumina HiSeq 2500. Subsequently, the raw reads were subjected to ACGT101-miR (LC Sciences, Houston, TX, USA) to remove repeats, junk, low complexity sequences, adapter dimers, and common RNA families (rRNA, tRNA, snRNA, and snoRNA). Subsequently, unique sequences ranging in length from 18 to 26 nucleotides were aligned to all mature mammalian miRNAs and their precursor sequences in miRbase 22.0 to identify known miRNAs using BLAST+ 2.9.0. Further, the remaining unmapped sequences that were matched with the sheep reference genome (Ovis aries v3.1) were used to predict novel miRNAs using miRDeep2. Differentially expressed (DE) miRNAs were analyzed based on normalized deep-sequencing counts using a *t*-test. The threshold for differential expression was set at *p* < 0.05.

Prediction of miRNA target genes was performed using TargetScan 50 and miRanda 3.3a software. A TargetScan Score > 50 and miRanda Energy < −20 were considered indicative of a targeting relationship. Further, miRNA target genes were subjected to GO and KEGG enrichment analyses. Statistical significance was set at *p* < 0.05.

### 4.5. mRNA and miRNA Data Validation Using qRT-PCR

A total of 14 DEGs and 11 DE miRNAs were randomly selected to validate the RNA-Seq data using real-time quantitative PCR. Reverse transcription for the first-strand cDNA was performed using the PrimeScript™ RT reagent kit with gDNA eraser (Perfect Real Time) (Takara Biotechnology, Dalian, China). The mRNA expression levels of genes were detected according to the manufacturer’s guidelines using TB Green™ Premix Ex TaqII (Takara Biotechnology, Dalian, China). Reverse transcription of the cDNA from miRNA was performed using the miRcute Plus miRNA First-Strand cDNA Kit (Tiangen Biochemical Technology, Beijing, China). The miRNA expression levels were detected according to the manufacturer’s guidelines using the miRcute Plus miRNA qPCR Kit (SYBR Green) (Tiangen Biochemical Technology, Beijing, China). GAPDH and U6 were used as internal reference genes to normalize mRNA and miRNA expression, respectively. Primer sequences are listed in Tables S1 and S2. All experiments were performed in triplicate for each biological replicate. Relative fold changes in target gene expression were calculated using the 2^−ΔΔCt^ method.

### 4.6. miRNA–mRNA Interaction Analysis

Using Cytoscape (version 3.10.1, Cytoscape Consortium), co-expression networks were constructed based on the DEGs and DE miRNAs identified in sheep SATs. We then determined co-expression relationships based on the negative correlation between miRNA and target gene expression, given the targeted interactions between miRNA and mRNA in different comparison groups.

## 5. Conclusions

This study investigated the dynamic changes in mRNA and miRNA profiles within SAT from naturally grazing Sunit sheep at 6, 18, and 30 months of age (n = 3). During sheep growth, changes in the expression of genes regulating lipid, fatty acid, glucose, and amino acid metabolisms and interactions with ECM receptors contributed to increased fat deposition and altered metabolites in Mth-18 and Mth-30 compared to those in Mth-6. Additionally, we discovered that *CAMKK2* and *PP2A* may inhibit the AMPK signaling pathway as sheep grow, potentially affecting the expression of genes related to lipid, glucose, and energy metabolism. Based on miRNA analysis, *mml-miR-320b*, *chi-miR-1388-3p*, *bta-miR-6715*, *oar-miR-143*, and *miR-424* may play dominant roles in SAT metabolism. Collectively, these findings elucidate critical genes and regulatory pathways involved in SAT development. We have also provided a theoretical basis for further breeding studies that may utilize gene editing technology to regulate expression profiles of these key genes to improve meat quality.

## Figures and Tables

**Table 1:** Pathways and gene expression trends associated with fat metabolism in Mth-18 compared to Mth-6.

Pathway id	Pathway Name	Upregulated Gene	Downregulated Gene
ko00100	Steroid biosynthesis	DHCR24, TM7SF2, CYP1B1, HSD17B12, STS	LIPA, SQLE, MSMO1
ko00061	Fatty acid biosynthesis	ACACA, FASN	ACSL4, ACSL5
ko01040	Biosynthesis of unsaturated fatty acids	ELOVL6, ENSOARG00000005352 (TECR), HSD17B12, SCD	ACOT7
ko00650	Butanoate metabolism	ACSM1, ACSM3, ACSM5, BDH1, HMGCLL1	
ko04152	AMPK signaling pathway	ACACA, FASN, PCK1, PFKFB4, PPARGC1A, PPP2R2B, SCD	HMGCR, PFKP, PIK3R3, PFKFB3
ko00051	Fructose and mannose metabolism	PFKFB4	HK3, PFKFB3, PFKP, TIGAR
ko00260	Glycine, serine, and threonine metabolism	ALAS2, GLYCTK	PSAT1
ko00062	Fatty acid elongation	ELOVL6, ENSOARG00000005352 (TECR), HSD17B12	ACOT7
ko00030	Pentose phosphate pathway	GLYCTK, GPI, TKT	PFKP
ko04514	Cell adhesion molecules (CAMs)	CNTN1, NFASC, NTNG1	CD274, CD86, PVR, SELE
ko00561	Glycerolipid metabolism	DGAT2, GLYCTK, GPAM	

**Table 2:** Pathways and gene expression trends associated with fat metabolism in Mth-30 compared to Mth-6.

Pathway id	Pathway Name	Upregulated Gene	Downregulated Gene
ko00561	Glycerolipid metabolism	DGAT2, DGKA, GLYCTK, GPAM, LPL	
ko04152	AMPK signaling pathway	ACACA, FASN, FBP2, HNF4A, PCK1, PFKFB4, SCD, SLC2A4	CAMKK2, FOXO3, PIK3R3
ko00650	Butanoate metabolism	ACSM1, ACSM3, ACSM5, BDH1, ECHS1	
ko00260	Glycine, serine, and threonine metabolism	ALAS2, GLYCTK, PHGDH	
ko01040	Biosynthesis of unsaturated fatty acids	ACAA1, ELOVL6, HSD17B12, SCD	
ko00030	Pentose phosphate pathway	FBP2, GLYCTK, RGN, TKT	
ko04010	MAPK signaling pathway	CSF1, FGF1, FGF2, FGFR2, GADD45G, PRKCA, RPS6KA2, RRAS2	DUSP10, JUN, KITLG, NR4A1, TAOK1, VEGFC
ko00062	Fatty acid elongation	ECHS1, ELOVL6, HSD17B12	
ko04151	PI3K-Akt signaling pathway	COL1A1, COL1A2, CSF1, FGF1, FGF2, FGFR2, FOXO3, LAMB3, PCK1, PIK3R3, THBS1, VEGFC	KITLG, NR4A1, PRKCA, SGK2

**Table 3:** Pathways and gene expression trends associated with fat metabolism in Mth-30 compared to Mth-18.

Pathway id	Pathway Name	Upregulated Gene	Downregulated Gene
ko04151	PI3K-Akt signaling pathway	CREB3L1, CSF1, ENSOARG00000005037 (THBS2S), ENSOARG00000011855 (ITGB3, CD61), FLT4, IL4R, ITGA5, RELN	ANGPT1, BRCA1, KIT, VEGFC, PPP2R2B
ko04010	MAPK signaling pathway	CSF1, DUSP4, FLT4, GADD45B, GADD45G	ANGPT1, KIT, VEGFC
ko04022	cGMP-PKG signaling pathway	CREB3L1	PDE3A, MYLK3
ko00051	Fructose and mannose metabolism	FBP2, HK3	
ko04014	Ras signaling pathway	CSF1, ENSOARG00000005737(RASSF5, RAPL), FLT4	ANGPT1, KIT, PLD1, VEGFC
ko04923	Regulation of lipolysis in adipocytes		PRKG1
ko0452	ECM–receptor interaction	CD44, ENSOARG00000005037 (THBS2S), ENSOARG00000011855 (ITGB3, CD61), ITGA5, RELN	

## Data Availability

The RNA-seq data were deposited to the NCBI SRA database (PRJNA1215330, PRJNA1217398). All data associated with this study are available by contacting the corresponding authors with a request.

## Supplementary Materials

The following supporting information can be downloaded at: https://www.mdpi.com/article/10.3390/ijms26073324/s1.
