# Supplementary material for: Mechanisms of Adipose Tissue Metabolism in Naturally Grazing Sheep at Different Growth Stages: Insights from mRNA and miRNA Profiles
Source: Int J Mol Sci. 2025 Apr 2;26(7):3324. doi: 10.3390/ijms26073324 (PMC11989906; doi:10.3390/ijms26073324)
Supplement: Supplementary file 1 [file ijms-26-03324-s001.zip › Supplementary Tables.pdf]

Table S1 mRNAs primer sequences

Gene	Primer
TKT	F:AGGTGGCATAGGTGAGGCAGTG
	R:GCGATGGCGTCCCTGTCAATG
THRSP	F:AGGCTGAGGAAGAGAGGGTATTGG
	R:GATGGGTGAGGATGTGATGAAGGC
ELOVL6	F:GCAAAGCACCCGAACTAGGAGATAC
	R:CAGGAGTACAGCAGCACAGTGATG
SDC4	F:GACGAGGATGTGTCCAACAAGGTG
	R:CGAGGAAGACGGCAAACAGGATG
ACSF2	F:CTCCAGTTACGACATCTCAACCATACG
	R:CCACCACTAGCTCCTTCATGTTCAG
GPI	F:GACCGCTTTAACCGCTTCAG
	R:TGCATCACAGTCTCCGTCAC
SCD	F:ACACTTGGGAGCGCTGTATG
	R:AGCCGAGCTTTGTAGGTTCG
FASN	F:AACGCTGTGGTGCTGGAGATTG
	R:GTTGTCCCTGTGGTCCTTCTTCATC
ACACA	F:GCTATGGAAGTCGGCTGTGGAAG
	R:CATTCGTCAGGAAGAGGCGGATG
DGAT2	F:CAACAGGTCCAAGGTAGAGAAGCAG
	R:ATGAGCCAGCAATCGGTACAGAATG
CAMKK2	F:GCCCGTTTCTACTTCCAGGATCTAATC
	R:CTTGAACTCATTGCTCACACCAAAGTC
CYP26B1	F:CAGGTGTCTTGGCAACCAGAGTG
	R:TTGTCGTGTGGCTGTGAATATCCG
COL24A1	F:CAAAGTTGGAGTTTGGAATCGGGAAAG
	R:GAGTCAATCCTGAGCCACTTGTCTG
PPP2R2B	F:TGAAGAGCCAGAAGATCCAAGCAAC
	R:TTCCATGTTGAGATCCCAGACTTTGAC
GAPDH	F:CGGCACAGTCAAGGCAGAGAAC
	R:CACGTACTCAGCACCAGCATCAC

Table S2 miRNA primer sequences

Gene	Primer
oar-miR-152	F：ACAGGCACGGCCAGTTTGA
pha-miR-106b_R+2	F：CCGCACTGTGGGTACTTGC
PC-5p-164_48170	F：CGTTTCCCGGCCAATGCA
ssc-mir-1285-p3_1ss24TA	F：CAACATAGCGAGACCCCGTCTCTA
bta-miR-339b_R+2	F：TCCCTGTCCTCCAGGAGC
hsa-miR-7977_1ss6AG	F：TATATATTCCCGGCCAACGCACCA
chi-miR-186-5P_R+1	F：CCGCAAAGAATTCTCCTTTTGGGCTT
chi-miR-145-5p	F：GTCCAGTTTTCCCAGGAATCCCT
chi-miR-140-3P	F：ACCACAGGGTAGAACCACGGA
chi-miR-1388-3p	F：ATAATCTCAGGTTCGTCAGCCCG
chi-miR-15a-5p_R-2	F：CGCTAGCAGCACATAATGGTTTGT
U6	F：GGAACGATACAGAGAAGATTAGC
	R：TGGAACGCTTCACGAATTTGCG

Table S3 Overview of mRNA sequencing data

Sample	Raw Data		Valid Data		Valid	Q20%	Q30%	GC%
	Read	Base	Read	Base				
Mth-6-1	94760134	14.21G	8944176	13.42G	94.39	99.98	97.90	44
Mth-6-2	89258604	13.39G	8490934	12.74G	95.13	99.98	97.83	44.50
Mth-6-3	91479924	13.72G	8640977	12.96G	94.46	99.98	97.84	44
Mth-18-1	96314016	14.45G	9083183	13.62G	94.31	99.98	97.99	44.50
Mth-18-2	82407182	12.36G	7750553	11.63G	94.05	99.98	97.98	45
Mth-18-3	91851066	13.78G	8584753	12.88G	93.46	99.98	98.01	44.50
Mth-30-3	89100230	13.37G	8404755	12.61G	94.33	99.98	97.99	44
Mth-30-2	95690878	14.35G	9015393	13.52G	94.21	99.98	97.93	45
Mth-30-3	81346720	12.20G	7765043	11.65G	95.46	99.99	98.71	45

Q20% and Q30% are the percentages of bases with phred values greater than 20 and 30 in the total bases respectively; GC% are the proportion of GC content

Table S4 Overview of miRNA sequencing data

Sample	Raw reads	valid reads
Mth-6-1	10180206	3545354
Mth-6-2	15235325	7473522
Mth-6-3	13295036	6478087
Mth-18-1	15913533	5855745
Mth-18-2	16895482	6038093
Mth-18-3	14615330	5927559
Mth-30-1	10197044	3960613
Mth-30-2	12850727	6332950
Mth-30-3	12834550	5592575
