# Supplementary figures and images for: Mechanisms of Adipose Tissue Metabolism in Naturally Grazing Sheep at Different Growth Stages: Insights from mRNA and miRNA Profiles
Source: Int J Mol Sci. 2025 Apr 2;26(7):3324. doi: 10.3390/ijms26073324 (PMC11989906; doi:10.3390/ijms26073324)

Length distribution of sequencing result (Total)

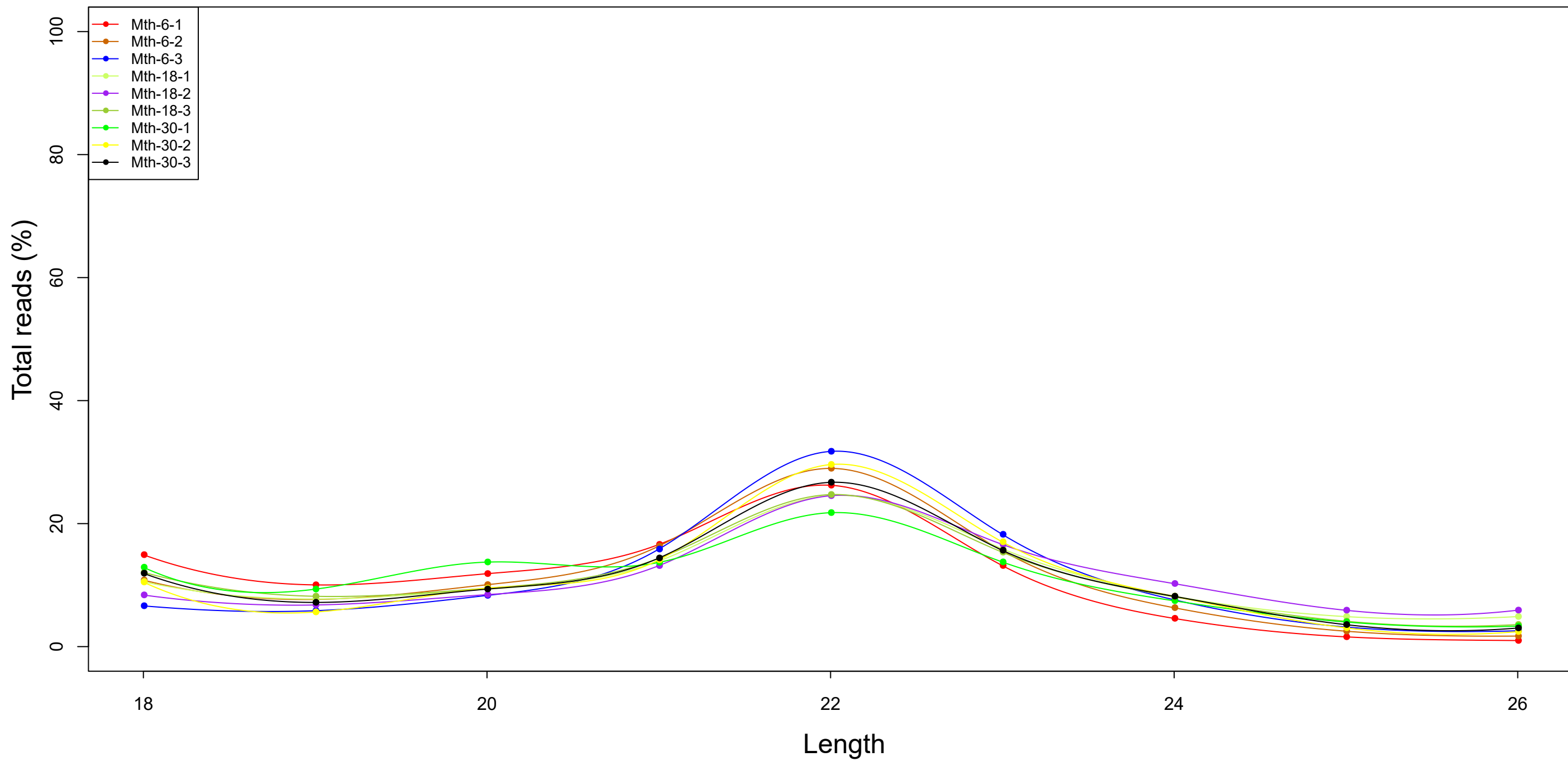

Supplement: Supplementary file 1 [file ijms-26-03324-s001.zip › Supplementary Figure S1.pdf]
